# Analysis of aflatoxins in traditional Chinese medicines: Classification of analytical method on the basis of matrix variations

**DOI:** 10.1038/srep30822

**Published:** 2016-08-04

**Authors:** Sheng-Ping Zhao, Dan Zhang, Li-Hong Tan, Bao Yu, Wei-Guo Cao

**Affiliations:** 1 College of Traditional Chinese Medicine, Chongqing Medical University, 400016, Chongqing, People’s Republic of China; 2 The Lab of Traditional Chinese Medicine, Chongqing Medical University, 400016, Chongqing, People’s Republic of China

## Abstract

A classification system for analytical methods was developed for the first time to determine the presence of aflatoxins B_1_, B_2_, G_1_ and G_2_ in traditional Chinese medicines (TCMs) based on different matrix types using ultra-performance liquid chromatography–tandem mass spectrometry. A useful characteristic of the approach was that the TCMs could be systematically divided into four categories (i.e., volatile oils, proteins, polysaccharides and fatty oils) depending on the matrix types. The approach concluded that different types of TCMs required different optimal sample preparation procedures. Based on the optimized analytical conditions, the limits of detection and quantification, average recoveries and linearity of four aflatoxins were determined and conformed to research limits. Of 22 TCMs samples, 14 samples were contaminated with at least one type aflatoxin at concentrations ranging from 0.2 to 7.5 μg/kg, and the average contents of aflatoxins were significantly different for the different matrix types. Moreover, we found a potential link between the contamination levels of aflatoxins and matrix types. TCMs containing fatty oils were the most susceptible to contamination by aflatoxins and followed by TCMs containing polysaccharides and proteins; TCMs containing abundant amounts of volatile oils were less prone to contamination.

Aflatoxins (AFs), namely aflatoxins B_1_ (AFB_1_), B_2_ (AFB_2_), G_1_ (AFG_1_) and G_2_ (AFG_2_), are secondary metabolites produced by fungal species, such as *Aspergillus flavus, Aspergillus parasiticus* and *Aspergillus nomius*^1^. AFs are carcinogenic, hepatotoxic, immunosuppressive, genotoxic, antinutritional, teratogenic and mutagenic to humans^2^,^3^,^4^ and AFB_1_ was defined as a Group 1A carcinogen by the International Agency for Research on Cancer (IARC)^5^. Due to the pernicious nature of AFs, many countries have established regulations to control the levels of AFs in food and agricultural products which are susceptible to fungal growth.

In China, traditional Chinese medicines (TCMs) with long histories of use are susceptible to mildew and fungus pollution and produce harmful mycotoxins during the production, processing, transportation and storage processes^6^. Therefore, China has formulated the following relevant standards: The limits for AFB_1_ and total AFs (sum of AFB_1_, AFG_1_, AFB_2_, and AFG_2_) in herbs and decoction pieces are 5 and 10 μg/kg, respectively (Chinese Pharmacopoeia, 2015). Other countries have established similar standards, the European Union in the Commission Regulation (EC) No. 1881/2006 has established the maximum residue limits (MRLs) of AFs: 2 μg/kg for AFB 4 μg/kg for the sum of the four AFs^7^. More than 1.5 billion people all over the world trust the efficacy and safety of TCMs^8^, and the daily consumption of TCMs is so huge. Hence our understanding of these materials should be strengthened to develop aflatoxin (AF) detection methods to ensure the safety of TCMs. Currently, detection methods exist for the monitoring of AF contamination in some TCMs^9^,^10^ such as licorice roots, fritillary bulbs, *Fructus Bruceae*, but comprehensive and systematic investigations on TCMs are lacking.

In recent years, many analytical techniques have been developed for the detection of AFs including thin layer chromatography (TLC)^11^, high performance liquid chromatography with fluorescence detector (HPLC-FD)^12^, iodine derivation after column(Chinese Pharmacopoeia 2015), enzyme-linked immunosorbent assays (ELISA)^13^,^14^ and high- (or ultra-) performance liquid chromatography-tandem mass spectrometry (HPLC-MS/MS or UPLC-MS/MS)^15^,^16^,^17^,^18^,^19^. UPLC-MS/MS methodologies with high resolution, high sensitivity and high selectivity have become a powerful tool for conducting research on complex chemical components^20^,^21^,^22^. The use of UPLC-MS/MS has been increasingly focusing on quantitative and qualitative analyses of traditional data obtained on AFs in TCMs.

Due to the complexity of TCMs, the matrix effect has become a main factor that has affected the accuracy of detecting AFs in TCMs^9^,^23^. Thus, the methods used for sample pre-treatment are very important for the accurate detection of AFs in TCMs. Sample pre-treatment mainly includes extraction and purification processes, and existing literature reports have shown that samples of different matrix types can be adopted using appropriate sample pre-treatment methods^9^,^18^,^23^,^24^. For instance, samples with fatty oils have high proportions of fatty oil contents; Huang B *et al*. adopted an extraction method using homogenization and a reliable solid phase extraction-based clean-up method to process such samples^25^. For cereal samples with high protein and polysaccharide (starch) contents, the extraction methods for AF samples frequently used ultrasonography^12^,^26^ and clean-up methods for AFs employed solid-phase extraction (SPE) methodologies^27^,^28^. The studies described above mostly utilized complex sample processing methods to accurately determine the presence of AFs in one or several samples. However, to accommodate the large number and variety of TCMs, much work is required to develop corresponding sample pre-treatment methods. If classified sample pre-treatment mode were to be established, the accuracy of the measurements and efficiency of work would improve.

Scholars have adopted classification methods^18^,^29^, and TCMs have always been divided into different medical parts, i.e., rhizomes, roots, seeds, flowers, grasses and leaves, which were extracted and purified using the same procedures depending on the medical parts. However, this classification method has some defects. For example, there may be a major difference in the matrix of the same medicinal parts of different medicinal materials. The same sample pre-treatment methods developed by such researchers were not suitable for extracting or detecting AFs in TCMs.

The aim of this work was to develop a novel classification of analytical method to detect aflatoxins B_1_, B_2_, G_1_ and G_2_ in widely applied TCMs based on different matrix types. Research efforts have focused on the influence of different sample pre-treatment methods on the samples and optimization of UPLC-MS/MS parameters. This research can offer a reference for systematically establishing analytical methods for the detection of AFs in TCMs. Meanwhile, data on the contamination levels of AFs and the contents of different matrix types were processed and analyzed using statistics software, and an inner relationship was found, which could be used to infer the susceptibility of fungus contamination based on the matrix types of TCMs and to provide a reference point for the safety of TCMs.

## Results and Discussion

### Analysis results of sample matrix types

The matrix types of all 22 TCMs were divided according to the contents of the basic components. The results are shown in Table 1. By comparing the content ratios of the four types of components in each sample, all 22 samples were divided into four matrix types, i.e., volatile oils, proteins, polysaccharides and fatty oils. In our work, the polysaccharide content was determined to be 2.2–31.8% in the 22 samples, among which the content of polysaccharides of five samples was larger than 20%; these five samples were eventually categorized into the polysaccharides group. Of the TCMs, 6 out of the 22 samples were classified as volatile oils, in which the range of the volatile oil content was between 1.6% and 2.9%. Similarly, fatty oil and protein contents ranged from 23.8% to 50.5% and from 15.9% to 21.3%, respectively, and these samples were categorized as the fatty oils and proteins, respectively.

Moisture contents of samples were tested based on the Chinese Pharmacopoeia (2015). The results were shown in Supplementary Table 1. For volatile oils, proteins, polysaccharides and fatty oils, the moisture content was 7.52–8.92%, 7.49–10.44%, 7.16–10.54%, and 5.37–7.13%, respectively. Moisture content results met the requirements of Chinese Pharmacopoeia.

### Optimization of the extraction procedure

**Figure 1:**
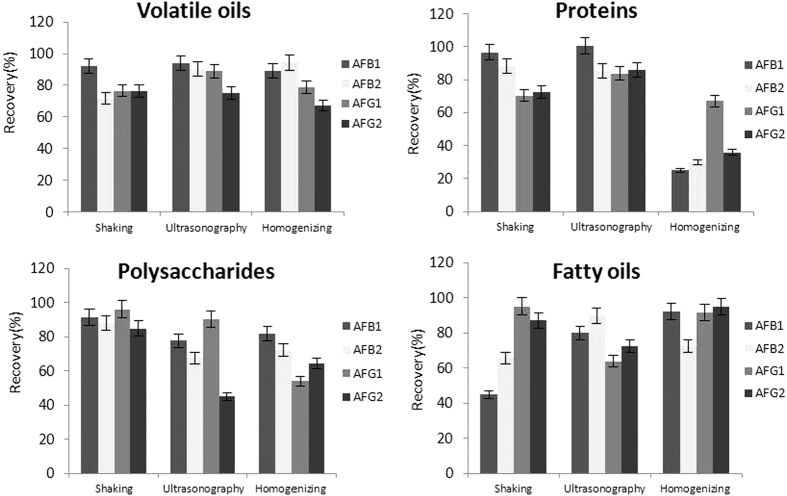
Efficiency of extraction for AFs in TCMs of different matrix types using different extract methods.

For the TCMs samples of four different matrix types, the effectiveness of various extraction methods was investigated. Four duplicate samples of the four types of matrices were extracted through shaking, homogenizing and ultrasonicating the samples. By comparing the extraction efficiencies of three methods, each sample of the four types of matrices required its own extraction methods (Fig. 1). Based on the results, ultrasonic extraction was selected as the best extraction method for the protein and volatile oil samples. Shaking extraction methods were determined to be the optimal methods for the samples of polysaccharides, and homogenization extraction was chosen for fatty oils. Because TCMs with high contents of fatty oils and polysaccharides were more viscous, an ultrasonography extraction method was prone to aggregating the extracts, and its use to extract AFs was not conducive to dissolution of the compounds.

In addition, to allow for higher extraction efficiencies, the extraction solvents and time were optimized. Five ratios of extraction solvents were investigated: 65%, 70%, 75%, 80%, and 85% aqueous methanol solutions were used for the samples of each type, and the samples were also subjected to different extraction times. The results of this optimization study are shown in Supplementary Fig. 1. For volatile oils, the samples were extracted in 75% aqueous methanol using ultrasonography for 45 min. The samples containing proteins were sonicated in 85% aqueous methanol for 45 min. For the samples with polysaccharides, they were extracted in 70% aqueous methanol for 3 h with shaking, and the samples of fatty oils were homogenized in 70% aqueous methanol for 4 min.

Because the samples had different matrices, each category of the samples required the use of a different extraction method. The obtained results were consistent with observations reported in previously published articles. A.S. Luna *et al*. conducted research on peanuts with more oil and used a homogenization extraction method to process the samples^30^. Wen J. *et al*. adopted an extraction procedure using ultrasonication to extract AFs from ginger and products related to volatile oils^31^. Kong W.J. *et al*. developed a method to analyse multi-class mycotoxins in Coix seeds^32^. However, in our work, shaking extraction was an optimal extraction method for samples containing polysaccharides.

### Optimization of the clean-up procedure

To optimize extraction efficiencies and the recovery of materials, different methods were tested and compared. In our study, the use of Welchrom C18E columns and silica gel columns for the clean-up procedures after extraction was evaluated. The first two methods were compared to samples that were not subjected to purification methods, which showed that the recovery_no purification_ > the recovery_C18 columns_ > the recovery_silicagel columns_ (Supplementary Table 2). Because the fatty samples contained more nonpolar and weakly polar compounds which could pollute and damage the UPLC column and consequently shorten the service life of the column upon purification, the samples needed to be processed after being subjected to a clean-up procedure. In general, the three types of TCMs mentioned above were extracted without purification, which resulted in a higher recovery rate and lower loss rate. Samples of fatty oils were purified by C18-SPE columns to protect the columns against damage, and the obtained recovery was 70–110% using the clean-up method and matched the recovery amount of the standard.

### Method validation

The ranges of linearity, the coefficients of determination and correlation, as well as the limits of detection (LOD) and quantification (LOQ) for each aflatoxin were determined. The working standard solutions of AFs were diluted immediately with methanol from the original stock solutions every weekday and which were used to make the mixed working standards. A set of four standard solutions containing different concentrations in the range of 0.0502–10.4 ng/mL for AFB_1_, 0.0350–7.0 ng/mL for AFB_2_, 0.0295–11.8 ng/mL for AFG_1_ and 0.0295–11.8 ng/mL for AFG_2_, which were prepared in methanol and were used for method calibration. These solutions were kept at −20 °C and were renewed weekly. The linearities obtained for all the analytes were good, and the correlation coefficients (*R*^*2*^) ranged from 0.9985 to 0.9996. LOD and LOQ values were 0.008–0.022 μg/kg and 0.011–0.029 μg/kg, respectively, which showed that the method developed, met the EU legislative requirements of 2 and 4 μg/kg for AFB_1_ and total AFs contents. The relative standard deviation (RSD) of precision at the middle concentration of the AFs mixture was 2.9–6.7% (n = 6). The data are shown in Table 2.

Recovery estimations were carried out using the standard addition method, which comprised three spiked samples at different levels. Different types of TCMs were used for the recovery test to ensure that the method had broad applicability. Each sample was selected at random, and aliquots (n = 9) of the samples were spiked with the mixed standard solutions at a high concentration level (10.4 ng/mL for AFB_1_, 3.5 ng/mL for AFB_2,_ 11.8 ng/mL for AFG_1 _and 5.9 ng/mL for AFG_2_), a medium concentration level (4.16 ng/mL for AFB_1_, 1.4 ng/mL for AFB_2,_ 4.72 ng/mL for AFG_1_ and 2.36 ng/mL for AFG_2_) and a low concentration level (1.04 ng/mL for AFB_1_, 0.35 ng/mL for AFB_2,_ 1.18 ng/mL for AFG_1 _and 0.59 ng/mL for AFG_2_). In general, a sample (2.0 g) was spiked with high, medium or low levels of the AF standards; and were treated and tested following the procedures outlined above. All recovery amounts ranged from 80.4% to 103.3% (Table 3). The spiked samples were extracted and analysed by UPLC-MS/MS, as previously described.

For the four AFs the results indicated good accuracy of the method for the detection of aflatoxins B_1_, B_2,_ G_1_, G_2_ in TCMs of different matrix types, and the recoveries were also in compliance with the requirements of the European Union (70–110%).

### Method application

Following the optimization and validation of the analytical approach, it was successfully utilized to determine the contamination levels of four AFs in 22 classified TCMs. The levels of total and individual AFs are summarized in Table 1.Typical UPLC–MS/MS chromatograms of the four AFs in standard solutions (A) and in contaminated samples (B) are shown in Supplementary Fig. 2. Of the 22 samples, 14 samples were detected to be positive with four AFs at concentrations ranging from 0.2 to 7.5 μg/kg, and 13 samples were detected to be contaminated with AFB_1_. The incidence rate was as high as 63.6%, and four positive samples (18.2%) exceeded the maximum limit set by the European Union (4 μg/kg). With regards to individual AFs, the levels of AFB_1_, AFB_2_, AFG_1_, and AFG_2_ were detected in ranges of 0.2–4.8, 0.1–2.3, 0.1–0.8, 0.1–0.2 μg/kg, respectively. For the four types of TCMs (i.e., volatile oils, proteins, polysaccharides and fatty oils), the levels of AFB_1_ were 0.2–0.4, 0.3–2.9, 1.4–3.2, 2.3–4.8 μg/kg, respectively, and the levels of AFs were 0.2–0.5, 0.4–3.5, 1.2–4.5, 3.8–7.5 μg/kg, respectively. Based on these results, we inferred that contamination of AFB_1_ was the most serious in the 22 TCMs samples.

### Correlation analysis

**Figure 2:**
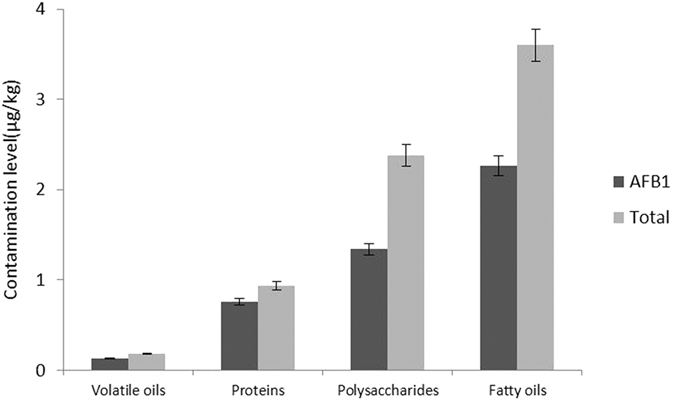
Average contamination levels of AFB_1_ and total AFs of samples from four matrix types.

To further analyse the contamination levels of the 22 TCMs, we compared the contents of AFB_1_ and AFs in the samples of four matrix types. The effects of the matrix types on the contamination levels of AFs are thought to be due to their different abilities for breeding fungus. The average contamination levels of AFB_1_ and total AFs in the samples of four matrix types are shown in Fig. 2. The content of AFs in the samples of different matrix types was varied significantly. Results showed that TCMs with an abundant of fatty oils had the highest amounts of AFB_1_ and total AFs, while these contamination levels were very low for samples with an abundance of volatile oils. Furthermore, the internal relation between the contamination levels of AFs and matrix types was studied.

In our study, the results obtained by Pearson correlation analysis indicated that the contents of AFB_1_ and total AFs had varying degrees of influence on the different matrices. As shown in Table 4, the contents of AFB_1_ and AFs were negatively correlated with the contents of volatile oils, and the correlation coefficients (*r*) were −0.612 and −0.556 (*P* < 0.05). respectively. The content of fatty oil exhibited a positive correlation to the contamination levels of AFB_1_ (*r* = 0.661, *P* < 0.01) and AFs (*r* = 0.749, *P* < 0.01). The contents of AFB_1_ and AFs were not significantly positively correlated with the contents of polysaccharides and proteins.

Our results indicate that TCMs with fatty oils may easily multiply *Aspergillus flavus* and *A. parasiticus*, resulting in the production of secondary metabolites (AFs). Polysaccharides and proteins also provided nutritional ingredients for fungus and promote their growth; the contents of AFs were relatively high in both types of TCMs. TCMs with volatile oils, such as *Fructus Tsaoko, Fructus Anisi Stellati* and *Flos Caryophylli* contain the active chemical components, known as essential oils, which possessed antifungal effects that reduced or prevented fungal infection and subsequent AFs production. The essential oils can decrease the damaged effect of aflatoxins by two different ways. Firstly, DNA binding formation of aflatoxins is reduced by essential oils. Secondly, aflatoxins cause increase of reactive oxygen species and essential oils react with reactive oxygen species. Therefore, essential oils protect the cells from harmful impact of aflatoxins^33^,^34^. Similar results have been reported for studies conducted on *Ocimum basilicum L.*^35^*, Radix Puerariae Lobatae* and *Semen Persicae* samples^18^.

## Conclusions

In this study, a classification method for the simultaneous detection of AFB_1_, AFB_2_, AFG_1_ and AFG_2_ in TCMs based on matrix types was established by UPLC-MS/MS for the first time, and the classification approach was successfully applied to analyse a total of 22 different matrix types of TCMs. This study provides a novel research approach for establishing the use of analytical methods to detect AFs in a large number of TCMs.

Furthermore, we found that there was significant relationship between matrix types and the contamination levels of AFs. The contents of fatty oils, polysaccharides and proteins to the contamination levels of AFB_1_ and AFs were positively correlated, whereas the contents of AFs were negatively correlated with the contents of volatile oils. Meanwhile, a possible association between the contamination levels of AFs and the different matrix types of TCMs was presented. The possibility for AFs contamination of medicinal materials containing fatty oils and polysaccharides was high, but the possibility of those containing volatile oils was low. These results indicate that the processing and storage methods used for medicinal materials are likely associated with the matrix types of their components, especially regarding the amounts of fatty oils of TCMs.

## Methods

### Materials and reagents

AF standards including AFB_1_, AFB_2_, AFG_1_ and AFG_2_ were purchased from Sigma-Aldrich (St. Louis, MO, USA). Solid powders of each aflatoxin standard were weighed accurately, and the standards were dissolved in methanol to prepare stock standard solutions and stored at −20 °C in a dark place. Distilled water was purified using a Milli-Q Gradient A 10 system (Millipore, Billerica, MA, USA). Acetonitrile, methanol and formic acid were of LC grade (Merck, Darmstadt, Germany). All the other solvents were of analytical grade. Welchrom C18E (500 mg/3 mL) columns were purchased from Welch (USA).

A total of 22 samples were randomly purchased from June to August 2014 from several local markets and drug stores in Chongqing China; the samples were authenticated by Professor Dan Zhang at Chongqing Medical University. All the samples were ground into powders, sieved through a 60-mesh filter and stored in sealed plastic bags below 4 °C for further analysis.

### UPLC-MS/MS analysis

The UPLC chromatography system (Shimadzu Corp., Kyoto, Japan) was equipped with a solvent delivery pump (LC-30AD), an auto-sampler (SIL-30AC) and a column oven (CTO-20AC). The separations were performed on a Phenomenex Luna 3 μC18 (2) 100A column (50 × 2.00 mm) (Phenomenex, USA). Chromatographic analyses were carried out using a gradient elution, where eluent A was an aqueous solution of ammonium formate (5 mM) and eluent B consisting of acetonitrile. The analysis started with 30% of acetonitrile, which was held for 0.5 min, and was then changed to 80% acetonitrile at 4.5 min and held 1.5 min. Then, the eluent was changed to 30% acetonitrile at 6.1 min. The column was conditioned with 30% acetonitrile for 1.9 min before the next injection. The flow rate was set at 0.35 mL/min, and the injection volume was 3 μL. Moreover, the column temperature was maintained at 30 °C.

Electrospray mass spectrometry (ESI-MS) was carried out using an API 4000 triple-quadrupole instrument from Applied Biosystems (AB Sciex, Framingham, MA, USA), equipped with an electro-spray ionization (ESI) source. The mass spectrometer was operated in positive ESI modes with multiple reaction monitoring (MRM) at unit mass resolution. Data acquisition and processing of the ESI-MS were obtained using Analyst^TF^ software (AB Sciex), and the accurate mass data for the molecular ions were processed by PeakView^TM^ 1.1.1 software (AB Sciex).The source/gas conditions were as follows: the curtain gases CAD and CUR were set at 4 and 25 psi, respectively. The ion source gas 1 (GS1) and ion source gas 2 (GS2) were set at 55 psi and 55 psi, respectively. The ionization source of the MS/MS detector had a capillary voltage of 5.5 kV, and the source temperature was set to 600 °C. The compound conditions were Entrance Potential (10.0) and Collision cell potential (12.0). The MRM transitions, applied cone voltages and collision energies are summarized in Table 2.

### Analysis of samples matrix types

To determine the matrix composition of various medicinal materials, the contents of volatile oils, proteins, polysaccharides and fatty oils of 22 samples were determined. The contents of these materials were determined according to the Chinese Pharmacopoeia (2015), the Kjeldahl determination method^36^, the phenol-sulfuric acid method^37^ and the Soxhlet extraction method^38^, respectively. The content ratios were then calculated to classify the samples according to the matrix types.

## Sample Preparation

### Extraction

To optimize the extraction procedure of AFs in TCMs, the influence of different extraction methods and factor levels based on the classification results of different matrix was investigated. (1) Extraction methods: For the TCMs of four matrix types, duplicate samples of each type were extracted through shaking, homogenization and ultrasonication. (2) Extraction solution: Five different ratios of extraction solvents were investigated: 65%, 70%, 75%, 80%, and 85% aqueous methanol solutions were used for samples of each type. (3) Extraction time: Samples of four matrix types were extracted for four different periods of time. Four different extraction procedures were then used for samples of different matrix types, which are described below:

Volatile oils: A 2 g portion of ground sample was soaked in 10 mL of a methanol/water (75:25, v/v) solution for 1 h and was sonicated for 45 min. The sample was then centrifuged at 3000 rpm for 5 min, and1 mL of the supernatant was filtered through a 0.22 μm syringe filter prior to analysis.

Proteins: A 2 g portion of ground samples was soaked in 10 mL of a methanol/water (85:15, v/v) solution for 1 h and was sonicated for 45 min, The following procedure was the same as that used for the extraction procedures for volatile oils.

Polysaccharides: A 2 g portion of a ground sample was extracted in 10 mL of a methanol/water (70:30, v/v) solution for 3 h by shaking the sample. The sample was then centrifuged at 3000 rpm for 5 min, and 1 mL of the supernatant was filtered through a 0.22 μm syringe filter prior to analysis.

Fatty oils: A 2 g portion of a ground sample was homogenized in 10 mL of a methanol/water (70:30, v/v) solution for 4 min and was centrifuged at 3000 rpm for 5 min. Then, 2 mL of the supernatant was subjected to the for clean-up procedure.

### Clean-up

To evaluate the efficiency of the clean-up procedure, results obtained using Welchrom C18E columns and silica gel columns were compared to samples that were not subjected to a purification method.

Samples of fatty oils were purified using the following procedure. A 2 mL aliquot of the final filtrate was passed through a Welchrom C18E column. The C18E column was pre-treated with 6 mL methanol before washing it with 6 mL distilled water. After the sample was loaded into the column, the column was first washed with 6 mL distilled water, and then the C18E column was rinsed with 4 mL methanol. The obtained elutes were completely evaporated under a steam of nitrogen gas at 30 °C, and the sample was re-dissolved in 1 mL methanol. The solution containing the AFs was vortexed for 30 s, and approximately 50 μL of the solution was filtered through a 0.22 μm filter. A 3 μL aliquot of the filtrate was injected into the UPLC-MS/MS system.

### Method validation

Quantification of the AFs in TCMs followed testing for linearity, recovery, LOD and LOQ. To check the linearity of the method, calibration curves based on the peak area were constructed in the range of 0.0295–11.8 ng/mL. To interpolate the results, concentrations outside the calibration range were performed with proper dilutions. Quantification was performed by plotting concentration versus peak area, and the regression curve was evaluated by using variance (ANOVA) analysis.

The LODs were obtained using a signal-to-noise ratio of S/N = 3:1, and the LOQ was considered the lowest point of the calibration curve that was adopted when the concentration of a compound resulted in S/N = 10:1.

Recovery analysis was performed by testing replicate spiked samples at three different concentrations (low, medium and high levels). The recovery values were estimated by relating the concentration of the AFs found to the expected concentration.

### Statistical treatment of data

To obtain further details of the differences, the UPLC-MS/MS datasets of the four groups were subjected to correlate analyses. The contents of AFB_1,_ total AFs (AFB_1_, AFB_2_, AFG_1_ and AFG_2_) and the content ratios of the four types of matrices were expressed as mean ± standard deviation of three replicates. The significance of each group was checked by a one-way analysis of Variance (ANOVA) followed by a Pearson correlation. A bivariate correlate analysis was used to determine the relationship between AFB_1_ contents, total AF contents and the content ratios of the four different types of matrices. Correlate analysis was conducted using SPSS 19.0 statistical software (SPSS Inc., Chicago, IL, USA). The significant value was set at *P* < 0.05.

## Additional Information

**How to cite this article**: Zhao, S.-P. *et al*. Analysis of aflatoxins in traditional Chinese medicines: Classification of analytical method on the basis of matrix variations. *Sci. Rep.* **6**, 30822; doi: 10.1038/srep30822 (2016).

## Supplementary Material

Supplementary Information

## Figures and Tables

**Table 1:** The content determination of volatile oils, fatty oils, polysaccharides and proteins in 22 TCMs, classification of samples matrix types, and the contamination levels of AFs in TCMs of different matrix types.

Category	Samples	The content ratio^a^ (%)				The contents^a^ (μg/kg)				
		Volatile oil	Protein	Polysaccharide	Fatty oil	AFB_1_	AFB_2_	AFG_1_	AFG_2_	Total^b^
Volatile oils	*Rhizoma Alpiniae Officinarum*	1.6	1.4	17.5	1.7	0.4	0.1	N.D	N.D	0.5
	*Fructus Anisi Stellati*	2.8	3.5	9.0	1.6	N.D	N.D	N.D	N.D	—
	*Fructus Citri Sarcodactylis*	2.2	6.4	13.4	3.0	0.2	0.2	N.D	N.D	0.4
	*Pericarpium Citri Reticulatae*	2.9	6.0	15.7	3.1	N.D	N.D	N.D	N.D	—
	*Fructus Tsaoko*	2.1	4.9	11.8	1.9	0.2	N.D	N.D	N.D	0.2
	*Flos Caryophylli*	2.3	5.1	15.6	3.7	N.D	N.D	N.D	N.D	—
Proteins	*Semen Phaseoli*	N.D	21.3	12.8	1.5	2.9	0.4	0.2	N.D	3.5
	*Semen Lablab Album*	N.D	17.0	9.0	0.8	0.6	0.2	N.D	N.D	0.8
	*Semen Coicis*	N.D	17.1	6.6	1.1	N.D	N.D	N.D	N.D	—
	*Semen Euryales*	N.D	15.9	2.2	2.2	N.D	N.D	N.D	N.D	—
	*Semen Nelumbinis*	N.D	19.7	5.6	1.8	0.3	0.1	N.D	N.D	0.4
Polysaccharides	*Fructus Mume*	N.D	3.1	26.8	1.4	1.4	0.4	0.1	N.D	1.9
	*Fructus Jujubae*	N.D	4.0	31.8	1.1	3.2	0.5	0.8	N.D	4.5
	*Fructus Hippophae*	N.D	10.4	21.8	1.8	N.D	N.D	N.D	N.D	—
	*Fructus Momordicae*	N.D	10.6	29.4	17.4	2.1	1.4	0.4	N.D	3.9
	*Fructus Rubi*	N.D	10.8	25.5	2.2	N.D	1.2	N.D	N.D	1.2
Fatty oils	*Semen Pruni*	N.D	14.6	15.0	39.2	2.7	1.4	0.3	N.D	4.4
	*Fructus Cannabis*	N.D	12.9	6.2	23.8	N.D	N.D	N.D	N.D	—
	*Semen Raphani*	N.D	14.0	15.6	37.7	3.8	1.2	0.1	N.D	5.1
	*Semen Armeniacae Amarum*	N.D	13.4	19.5	43.9	4.8	2.3	0.3	0.1	7.5
	*Fructus Perillae*	N.D	14.9	2.2	46.3	N.D	N.D	N.D	N.D	—
	*Semen Sesami Nigrum*	N.D	11.8	7.6	50.5	2.3	1.0	0.3	0.2	3.8

N.D not detected.

^a^Mean ± SD, n = 3.

^b^The sum of AFB_1_, AFB_2_, AFG_1_ and AFG_2_.

**Table 2:** ESI-MS/MS parameters, concentration ranges (ng/mL), limits of detection (LOD), limits of quantification (LOQ) and linearity values (*R* ^*2*^) for AFs.

AFs	MW	Q1 (m/z)	Q3 (m/z)	CE (e/V)	DP (V)	range (ng/mL)	*R*^*2*^	LOD (μg/ kg)	LOQ (μg/kg)	RSD (%)
AFB_1_	312.3	313.3	285.3^a^	30	178	0.0502–10.4	0.9987	0.008	0.011	2.9
		313.3	241.0	47	166					
AFB_2_	314.3	315.3	287.1^a^	33	161	0.0350–7.0	0.9992	0.015	0.023	3.5
		315.3	259.0	38	159					
AFG_1_	328.3	329.2	311.2^a^	30	143	0.0295–11.8	0.9985	0.022	0.029	4.6
		329.2	243.1	34	158					
AFG_2_	330.3	331.2	217.0^a^	46	131	0.0295–11.8	0.9991	0.020	0.027	3.4
		331.2	245.3	38	114					

^a^Quantitative ion.

**Table 3:** Recovery results of AFB_1_, AFB_2_, AFG_1_ and AFG_2_ a (%).

Category	Samples	Levels	AFB_1_	AFB_2_	AFG_1_	AFG_2_
Volatile oils	*Rhizoma Alpiniae Officinarum*	Low	89.4	87.3	90.4	85.6
		Medium	91.4	88.0	96.2	84.9
		High	90.3	82.0	95.1	87.1
	*Fructus Anisi Stellati*	Low	90.9	84.2	89.4	88.8
		Medium	94.4	96.7	100.5	85.2
		High	85.4	84.9	86.1	89.9
	*Fructus Citri Sarcodactylis*	Low	93.6	82.3	86.8	83.7
		Medium	87.0	92.3	100.1	96.2
		High	83.7	88.2	85.8	84.0
	*Pericarpium Citri Reticulatae*	Low	91.4	94.3	91.5	91.4
		Medium	90.9	89.6	84.8	91.3
		High	82.6	85.9	97.6	86.5
	*Fructus Tsaoko*	Low	95.8	92.3	82.4	81.2
		Medium	100.3	84.7	92.7	84.3
		High	81.3	94.3	86.5	86.2
	*Flos Caryophylli*	Low	96.4	90.0	88.4	93.9
		Medium	81.7	83.9	91.4	100.6
		High	93.7	89.7	89.4	85.9
Proteins	*Semen Phaseoli*	Low	84.6	101.1	90.8	93.7
		Medium	100.2	97.9	88.1	89.0
		High	101.2	84.4	86.3	90.4
	*Semen Lablab Album*	Low	83.4	84.7	81.2	83.4
		Medium	91.1	82.2	101.0	80.6
		High	82.1	81.1	92.7	84.2
	*Semen Coicis*	Low	87.9	91.6	80.4	96.1
		Medium	97.1	98.3	90.8	89.2
		High	88.1	98.5	92.1	96.5
	*Semen Euryales*	Low	82.8	95.3	100.5	99.4
		Medium	85.6	85.0	88.6	100.0
		High	83.0	95.7	80.9	92.4
	*Semen Nelumbinis*	Low	91.4	91.2	87.6	94.2
		Medium	84.9	89.2	92.8	85.4
		High	89.7	100.1	86.4	84.7
Polysaccharides	*Fructus Mume*	Low	83.4	93.6	81.2	83.4
		Medium	91.1	82.2	101.4	80.6
		High	102.2	95.6	94.6	93.4
	*Fructus Jujubae*	Low	91.4	93.8	100.1	99.7
		Medium	95.8	83.5	100.5	81.3
		High	97.7	92.9	88.0	98.8
	*Fructus Hippophae*	Low	88.3	83.3	100.6	91.3
		Medium	87.6	83.0	102.8	80.4
		High	80.8	90.6	84.4	93.4
	*Fructus Momordicae*	Low	96.7	101.2	90.5	101.0
		Medium	84.5	86.3	86.4	99.4
		High	88.3	91.9	84.2	88.7
	*Fructus Rubi*	Low	103.1	90.5	85.6	88.0
		Medium	97.5	87.4	98.4	90.3
		High	93.4	88.0	91.9	81.2
Fatty oils	*Semen Pruni*	Low	91.1	101.5	90.2	84.6
		Medium	83.6	92.5	81.7	82.0
		High	94.0	89.3	92.4	84.5
	*Fructus Cannabis*	Low	88.2	82.9	88.7	86.1
		Medium	91.4	98.3	98.4	81.9
		High	90.7	82.4	94.0	82.1
	*Semen Raphani*	Low	90.6	84.4	86.5	85.3
		Medium	88.4	97.9	100.2	90.7
		High	86.5	81.8	82.6	86.9
	*Semen Armeniacae Amarum*	Low	83.3	92.8	97.0	99.7
		Medium	83.9	86.3	93.4	99.4
		High	88.3	91.9	84.2	88.7
	*Fructus Perillae*	Low	102.1	90.5	85.6	88.0
		Medium	97.5	87.4	98.4	90.3
		High	93.4	88.0	91.9	81.2
	*Semen Sesami Nigrum*	Low	84.6	101.1	90.8	93.7
		Medium	100.2	97.9	88.1	89.0
		High	101.2	84.4	103.3	90.4

^a^Each value represents the mean ± SD of at least three measurements.

**Table 4:** The correlation between the contents of volatile oils, fatty oils, polysaccharides, and proteins in AFB_1_ and total AFs.

Component	Volatile oil	Protein	Polysaccharide	Fatty oil
AFB_1_	*r* = −0.612*	*r* = 0.266	*r* = 0.361	*r* = 0.661**
AFs	*r* = −0.556*	*r* = 0.240	*r* = 0.289	*r* = 0.749**

^**^extremely significant, *P* < 0.01;

^*^significant, *P* < 0.05.
