# Supplementary material for: Analysis of aflatoxins in traditional Chinese medicines: Classification of analytical method on the basis of matrix variations
Source: Sci Rep. 2016 Aug 4;6:30822. doi: 10.1038/srep30822 (PMC4973246; doi:10.1038/srep30822)
Supplement: Supplementary Information [file srep30822-s1.pdf]

Supplementary Information

Analysis of aflatoxins in traditional Chinese medicines: Classification of analytical method on the basis of matrix variations

Sheng-Ping Zhao, Dan Zhang, Li-Hong Tan, Bao Yu, Wei-Guo Cao

Supplementary Table 1. Moisture content results of 22 TCMs.

Supplementary Table 2. Recovery results of three purification methods.

Supplementary Figure 1. Efficiency of extraction for AFs in TCMs of different matrix types using different extract solvents (A) and extraction times (B).

Supplementary Figure 2. MRM chromatograms of aflatoxin standards (A) and the positive sample (B).

Supplementary Table 1. Moisture content results of 22 TCMs.

Category	Samples	Moisture content[a] (%)
Volatile oils	*Rhizoma Alpiniae Officinarum*	8.92
	Fructus Anisi Stellati	7.52
	Fructus Citri Sarcodactylis	8.41
	Pericarpium Citri Reticulatae	8.23
	Fructus Tsaoko	7.85
	Flos Caryophylli	8.07
Proteins	*Semen Phaseoli*	10.44
	Semen Lablab Album	7.49
	Semen Coicis	8.69
	Semen Euryales	9.33
	Semen Nelumbinis	9.29
Polysaccharides	*Fructus Mume*	7.16
	Fructus Jujubae	10.54
	Fructus Hippophae	7.92
	Fructus Momordicae	7.65
	Fructus Rubi	8.91
Fatty oils	*Semen Pruni*	7.13
	Fructus Cannabis	7.26
	Semen Raphani	5.53
	Semen Armeniacae Amarum	5.82
	Fructus Perillae	6.58
	Semen Sesami Nigrum	5.37

[a] Each value represents the mean ± SD of at least three measurements.

Supplementary Table 2. Recovery results of three purification methods.

Aflatoxins	Recovery (%)[a]		
	No purification	C18 columns	Silicagel columns
AFB_1	103.4(5.3)	95.6(6.1)	90.3(5.4)
AFB_2	96.7(5.2)	93.1(6.8)	92.5(4.3)
AFG_1	99.2(7.2)	93.3(4.9)	86.9(6.7)
AFG_2	95.8(4.4)	93.7(3.1)	83.1(5.9)

[a] RSD is given in brackets (n=3).

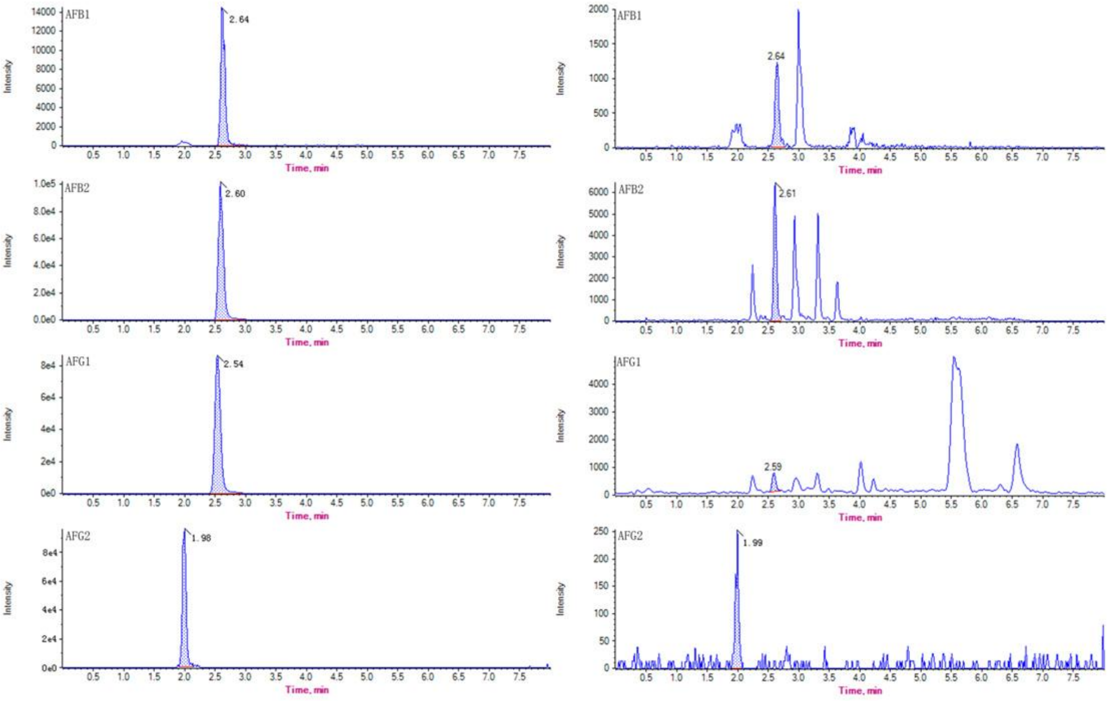

A B

Supplementary Fig. 1. Efficiency of extraction for AFs in TCMs of different matrix types using different extract solvents (A) and extraction times (B).

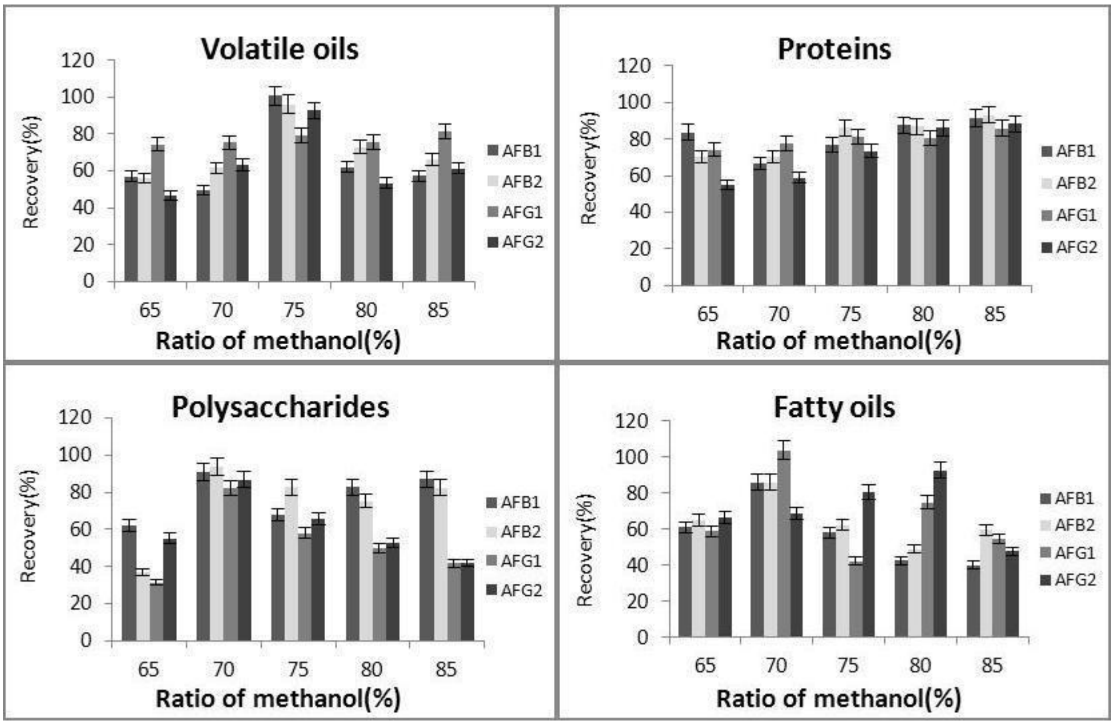

A

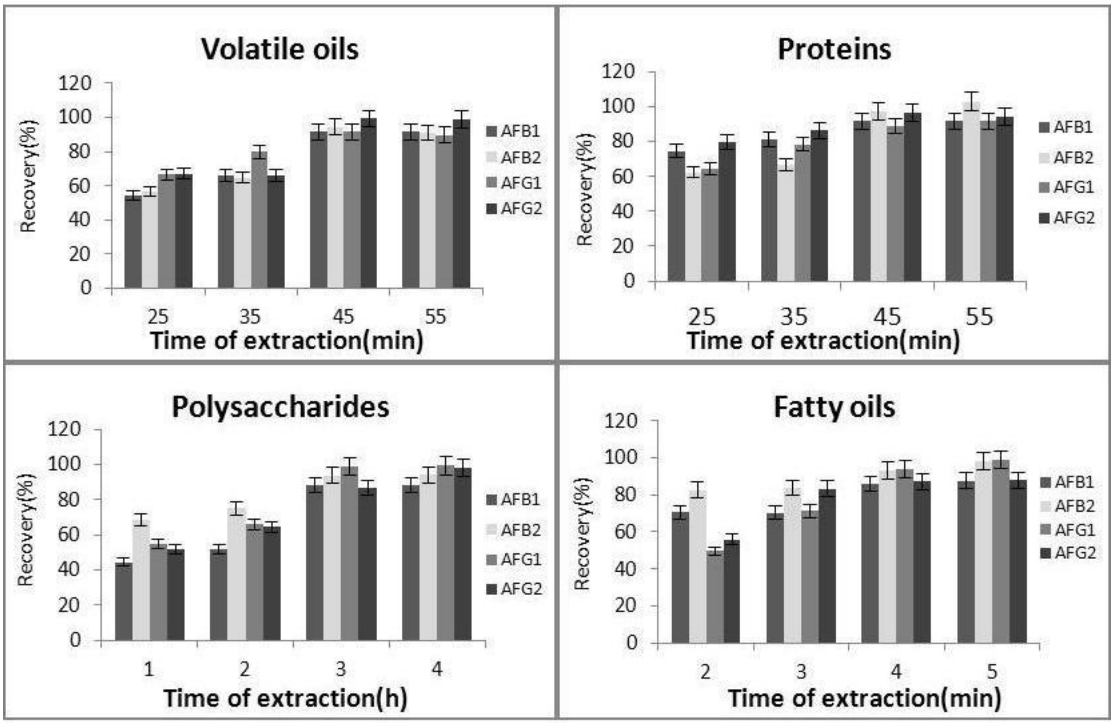

B

Supplementary Fig. 2. MRM chromatograms of aflatoxin standards (A) and the positive sample (B).
